# Evaluation of skills lab communication training in a bachelor’s programme in psychology based on communicative self-efficacy

**DOI:** 10.3205/zma001746

**Published:** 2025-04-15

**Authors:** Maren Menzel, Swetlana Philipp, Katrin Schulz, Thomas Fankhänel, Sabine Rehmer, Susanne Hardecker, Dorothea Portius, Sara Ramminger, Ulrike Zergiebel, Maximilian Schochow, Hiltraut Paridon, Sylvia Sänger, Anja Trummer, Alexander Ernst, Sandra Meusel, Katharina Wick

**Affiliations:** 1 SRH University of Applied Science Heidelberg, Campus Gera, Gera, Germany; 2 University Hospital Jena, Institute for Psychosocial Medicine, Psychotherapy and Psychooncology, Jena, Germany; 3 Baden-Württemberg Cooperative State University (DHBW), Heilbronn, Germany; 4 Martin-Luther-University Halle-Wittenberg, Halle/Saale, Germany; 5 Fresenius University of Applied Sciences, Idstein, Germany; 6 Nordhausen University of Applied Sciences, Nordhausen, Germany; 7 Gera-Eisenach Cooperative University, Gera, Germany

**Keywords:** communicative self-efficacy, communication training, simulation-based training, simulated patients, skills lab, practice-based studies

## Abstract

**Aim:**

The aim of this study is to test and evaluate a one-time communication training session.

**Methods:**

The focus of the evaluation is on the self-assessed communicative self-efficacy (SE-12) of students in the undergraduate degree programme in psychology before and after completing the communication training in the skills lab, during which simulated patients were used. The communication training was also evaluated by the students, and both positive feedback and suggestions for improvement were recorded. To do this, a quantitative study with a quasi-experimental design and a retrospective pre-post measurement (then-test) was conducted. The sample consists of 16 students in the undergraduate psychology programme at the SRH University of Applied Sciences Campus Gera (age: *M*=20.9, *SD*=1.7; 87.5% female). The available data was analysed descriptively and using t-tests.

**Results:**

The results show that students who completed the communication training report significantly higher communicative self-efficacy than prior to the communication training (t(15)=-6.04, p<.001, d=.75). Furthermore, the students rated the communication training positively.

**Conclusion:**

From the results, it can be concluded that simulation-based communication training can positively influence psychology students' communicative self-efficacy and that they rate the training positively. Communication training can be viewed as an important means to implement the mandated teaching of professionally relevant skills to psychology students in order to prepare them for professional participation in the labour market.

## Zusammenfassung

**Zielsetzung:**

Ziel der vorliegenden Arbeit ist es, ein einmaliges Kommunikationstraining zu erproben und zu evaluieren.

**Methodik:**

Der Fokus der Evaluation liegt bei der selbsteingeschätzten kommunikativen Selbstwirksamkeit (SE-12) von Studierenden im Bachelorstudiengang Psychologie vor und nach der Absolvierung eines Kommunikationstrainings im Skills-Lab, bei welchem Schauspielpatient:innen zum Einsatz kommen. Weiterhin soll das Kommunikationstraining von den Studierenden bewertet und sowohl positive Aspekte als auch Verbesserungsvorschläge erfasst werden. Dazu wird eine quantitative Studie mit quasi- experimentellem Design und einer retrospektiven Prä-Post-Messung (Then-Test) durchgeführt. Die Stichprobe besteht aus 16 Studierenden des Bachelorstudiengangs Psychologie an der SRH Hochschule für Gesundheit (Alter: *M*=20,9, *SD*=1,7; 87,5% weiblich). Die vorliegenden Daten werden sowohl deskriptiv als auch mithilfe von t-Tests ausgewertet.

**Ergebnisse:**

Die Ergebnisse zeigen, dass Studierende, die ein Kommunikationstraining absolviert haben, eine signifikant höhere kommunikative Selbstwirksamkeit angeben als vor dem Kommunikationstraining (*t*(15)=-6,04, *p*<.001, *d*=.75). Zudem wird das Kommunikationstraining von den Studierenden positiv bewertet.

**Schlussfolgerung:**

Aus den Ergebnissen lässt sich für diese Durchführung ableiten, dass simulationsbasierte Kommunikationstrainings einen positiven Einfluss auf die kommunikative Selbstwirksamkeit von Psychologiestudierenden haben können und positiv eingeschätzt werden. Ein Kommunikationstraining kann als wichtiger Beitrag zur geforderten Vermittlung berufsrelevanter Kompetenzen bei Psychologiestudierenden angesehen werden, um sie auf die berufliche Tätigkeit auf dem Arbeitsmarkt vorzubereiten.

## 1. Introduction

Communication skills are described as a set of indispensable key competencies for successful conversations and as a central basis for all actions undertaken in the healthcare professions [1], [2]. The ability to communicate professionally makes it possible to steer conversations competently, to assess a situation appropriately, and to express one’s own intentions verbally and non-verbally [3], [4]. Hence, communicative competence in holding conversations can be seen as an important tool for psychologists and psychotherapists [5]. The German Psychological Society [6] ascribes particular importance to the teaching of soft skills, such as conversational techniques, and recommends exploring the possibilities for teaching these skills in every type of course in undergraduate and graduate degree programmes. One particularly practical option is simulation-based training. This is already an integral part of many medical school curricula [7], although it is described as new and innovative in the teaching of clinical psychology and psychotherapy [8].

### 1.1. Simulation-based training

In vocational training, simulations emulate tasks, abilities or skills that are required for practical work [9]. Competencies in different areas can be trained and evaluated [8], [9], [10]. The implementation of simulation-based training usually takes place in skills labs, which are specially equipped training facilities where practical vocational skills and abilities can be taught and practised in a standardised manner [11]. Skills labs provide a safe and error-tolerant environment in which clinical skills can be practised in various scenarios without putting patients at risk [12], [13]. The spatial and technical requirements of a skills lab, as well as information on preparation and curricular implementation, are described in detail by Pierre & Breuer [14]. In simulation-based training, practical skills such as diagnostic, interventional and surgical techniques are realistically demonstrated and trained with the help of simulation manikins of premature newborns, infants and adults [14]. It is also possible to simulate various dialogues and illnesses using simulated patients so that students acquire and solidify interpersonal and communication skills early on in a degree programme alongside their technical skills [8], [14], [15], [16]. To this end, lay or professional actors are selected and trained to take on specific patient roles in a conversational context [16], [17]. It is also possible to speak of standardised patients here, since the same behavioural patterns, symptoms, personality traits and communication styles are portrayed in every simulation [12], [18]. Although actors can also portray relatives or colleagues, this rarely takes place in teaching, which is why we can generally speak of simulated patients (SP) [17]. While SP have been an integral part of the medical curricula in various teaching and examination scenarios internationally [19] and across Germany, ever since the 2002 medical licensure regulations (ÄApprO) came into effect [14], [17], they are used to a much lesser extent in psychology [8]. Non-standardised role plays have mainly been used to date at universities to teach psychology. One disadvantage of this method is that students usually have to slip into their role spontaneously without prior training, which means that there is a great deal of variability regarding the design. Moreover, the students find themselves in a familiar social environment which can lead to less authentic behaviour. This means that simulation in the form of role-playing is only suitable to a limited extent for therapeutic conversations [20]. Yu et al. [21] show that medical students reported lower levels of anxiety and higher levels of confidence in patient communication and physical examination after receiving simulation training than they did before the training. In another survey of medical students, 96.7% rated the use of SP in a course on anamnesis as practical and helpful [22]. This was also confirmed by Eckel et al. [23], who show that medical students predominantly rated the use of SP to train communicative competencies in the subjects of psychotherapy, medical psychology, psychosomatics, and psychiatry as authentic and positive, and that the students perceived a subjective learning gain. Training in communication skills can also have a positive effect on the self-efficacy of professionals [24]. Several studies have described the particular effectiveness of simulation-based training programmes in regard to the self-efficacy of communication skills in healthcare professionals [24], [25], [26].

### 1.2. Aim

In response to the revised German Psychotherapy Act (PsychThG) of 2020, greater emphasis is now placed on practice-based undergraduate education in clinical psychology [27], in which therapeutic skills can be acquired using SP. The German Psychotherapy Act also allows for the use of SP in practical multi-station exams at the end of the master’s degree programme in clinical psychology and psychotherapy [27]. Brakemeier et al. [28] have already trialled the use of SP as part of a multi-station exam for psychotherapeutic licensing. Alpers et al. [20] investigated the effect of using SP in the counselling practicum in the master’s degree programme in clinical psychology and psychotherapy on self-perceived therapeutic competence. Increased therapeutic self-efficacy was found after a two-day block seminar. However, Alpers et al. [20] leave open the questions of how many sessions are needed to increase self-efficacy and what standards are required for the use of SP in a graduate psychotherapy programme. To test whether the early, one-time use of simulation-based training in the bachelor’s programme has a positive effect on the communicative self-efficacy of psychology students, a skills lab station was developed and evaluated in a pilot study. In addition, we analysed how students assess the skills lab communication training so as to make adjustments based on their feedback and implement additional stations in the curriculum.

## 2. Methods

### 2.1. Procedure

**Figure 1:**
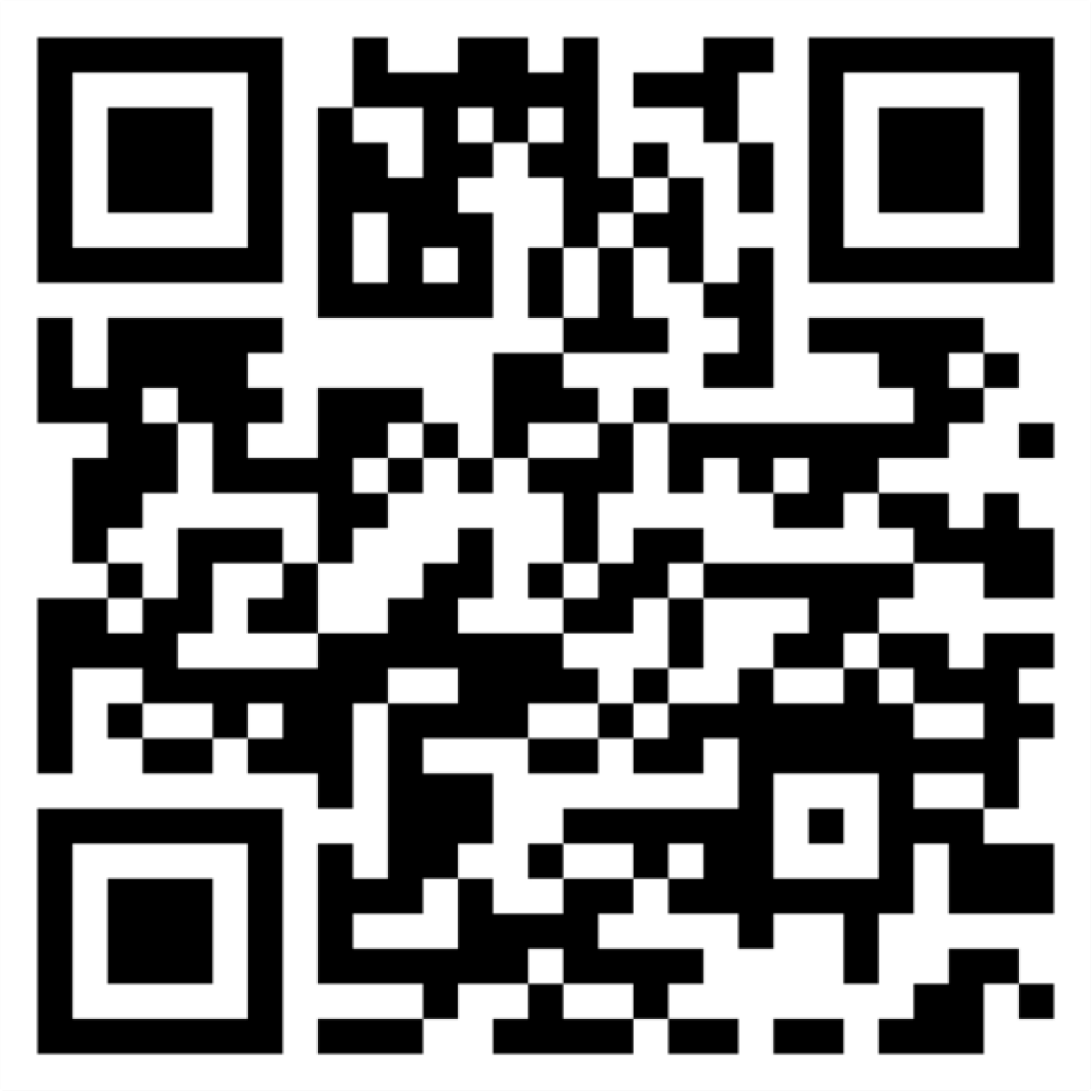
QR code for training materials and implementation

As part of this pilot study to set up the first skills lab station at the SRH University of Applied Sciences Campus Gera, a 60-minute communication training session was developed for the module on “clinical interventions” in the undergraduate psychology programme. There was previously no skills lab at the SRH University of Applied Sciences Campus Gera. As the spatial requirements for communication training are minimal, a room at the university was converted into a single communication training station. The communication training was intended to enable students to practise a conversation with a depressive patient in an inpatient rehabilitation facility and to receive feedback in order to increase their communicative self-efficacy. The training strategy included role instructions, a persona and a feedback sheet for the SP, as well as an assignment for the students. A feedback sheet and a room checklist were also designed for the tutor (sixth-semester psychology student). These are previously tried and tested materials that were developed based on existing communication situations. The feedback sheet serves as a guide during feedback and not as a fixed assessment measure. These materials can be found via the QR code in figure 1 (Fig. 1) or are available under [https://me-qr.com/CRjKH1m4] and were presented and integrated in the training of the SP. It should be noted that the materials need to be tailored to each station and for each role, as well as for any detailed training (see attachment 1 , only in German).

### 2.2. Process

The communication training materials were developed between October 2022 and March 2023. Four amateur actresses who were not part of the student cohort were recruited for the role of the SP. These SPs and the communication training tutor received full-day training from an employee of the Institute for Psychosocial Medicine, Psychotherapy and Psychooncology at the Jena University Hospital, where SP have been trained for the skills lab and other courses since 2011. The tutor was also trained to guide the communication training situation and provide support if the students were unsure. Furthermore, there was feedback training and a final rehearsal of the interview, during which all SPs and the skills lab manager were present to provide feedback.

One week before the communication training, the fourth-semester students attended the introductory lecture in the “clinical interventions” module. Initial instructions were given regarding the timeframe and content requirements for the communication training, which then took place from 16-23 May 2023 as a compulsory module session. The students took part in the communication training individually and only in the presence of the SP and the tutor, for which a period of 60 minutes was planned. The student's task was to inform the patient about the existing clinical picture of depression using the model of depression for behavioural activation by Martell et al. [29]. After familiarising themselves with the assignment, the student was given 20 minutes to complete the task. Two minutes before this time expired, the student was given a time card by the tutor. The student was then asked to leave the training room so that the SP could make notes for the feedback. This was followed by structured feedback from the SP and the tutor to the student in the training room (15 minutes). The remaining time was used to prepare the training room and for brief consultations between the tutor and the SP. After all of the students had completed the communication training, the evaluation was conducted online between 30 May 2023 and 22 June 2023 at a location of the students’ choice. Students were invited to take part in the survey voluntarily within a period of five days to five weeks after the training (on average 7.9 days). In addition, students received half a credit toward time spent as a trial subject if they completed the survey.

### 2.3. Sample

A total of N=18 students from the bachelor's programme in psychology at the SRH University of Applied Sciences Campus Gera completed the communication training course. Of these, N=16 students took part in the survey (response rate: 89%). The age of the students ranged between 19 and 25 years (*M*=20.9, *SD*=1.7). Of the N=16 students, 14 (87.5%) were female and 2 (12.5%) were male. One student was in their third semester and 15 students were in their fourth semester.

### 2.4. Questionaire

The questionnaire used to evaluate the training session can be divided into three sections, with the first part recording the students’ demographic data. The second section corresponds to the version of the Self-Efficacy Questionnaire (SE-12) according to Axboe et al. [30], translated into German by the author, and was administered twice to record communicative self-efficacy both before completion (pre) by means of a retrospective assessment and after completion (post) of the communication training at a measurement point in time (then-test). All items for measuring communicative self-efficacy included a 10-point response scale ranging from 1=“very uncertain” to 10=“very confident” (example item: How confident were you that you could successfully listen attentively without interrupting or changing focus?) The higher the values, the better the students rated their communicative self-efficacy. The overall scale was analysed as an overall mean value (scale range 1-10) and achieved a good or very good Cronbach's alpha of .80 (pre) and .92 (post). The last section of the questionnaire relates to the assessment of individual aspects of the communication training in the skills lab, for example, whether the assignment corresponded to the level of knowledge or whether the feedback from the SP was helpful. This section comprises a total of 14 items, which were developed based on the evaluation form used by the Jena skills lab. The items were assessed on a five-point, Likert-type response scale. The values ranged from 1=“completely inaccurate” to 5=“completely accurate” (example item: The communication course was appropriate to my level of knowledge.). Higher values indicate a more positive assessment of the communication training. A negative evaluation was assumed if a value <3 was given. For the overall assessment of the training, the values of the individual items were totalled and the average was calculated (scale range 1-5). The Cronbach’s alpha for the scale for this sample was .94 and can therefore be rated as excellent. The questionnaire also contained two boxes for free text responses concerning positive aspects of the skills lab station or comments and suggestions for improvement.

### 2.5. Data analysis

The quantitative data analysis was carried out using the statistical programme SPSS (version 27.0.1.0, IBM Corp.). A t-test for dependent samples was calculated to determine possible differences in the assessment of self-efficacy (overall score) before and after the communication training. Due to the exploratory nature of the study, a false discovery rate (FDR) correction was applied to correct for multiple testing. The requirement for normal distribution was tested using the Kolmogorov-Smirnov test for the overall mean value and was satisfied. In addition, the mean values were compared at the level of individual items. Visual inspection of the individual difference values in the Q-Q plot showed an approximately normal distribution (with a few exceptions, all points were on the straight line). All statistical analyses were performed with a confidence interval of 95% and taking into account an alpha error probability of 5%. The effect size of the t-test was given as Cohen’s d [31]. Here, d≥0.2 corresponds to a small effect, d≥0.5 to a medium effect, and d≥0.8 to a large effect. In addition, the maximum, average and minimum values of the students’ assessments of the skills lab station were recorded and the feedback was analysed descriptively. The evaluation of the free text responses was summarised in clusters as suggested by Mayring & Fenzl [32].

## 3. Results

### 3.1. Communicative self-efficacy

With regard to self-assessed communicative self-efficacy, a significant difference was found between the total score before the communication training (*M*=6.79; *SD*=1.11) and after the communication training (*M*=7.93; *SD*=1.24) (t(15)=-6.04, p<.001, d=.75), in that the scores improved from the retrospective pre- to post-measurement. This is a medium effect. All other statistically relevant parameters at the level of individual items are presented in table 1 (Tab. 1).

### 3.2. Assessment of the communication training

Overall, the communication training was evaluated positively by the students (*M_Total_*=4.05; *SD*=1.02). Four out of 14 items (28.57%) were rated neutral to positive (M≥3). Six items received a rating between 2 and 5 (42.86%); four items received a rating between 1 and 5 (28.57%). The students gave the highest ratings to the item asking if the tutor appeared competent (*M*=4.73; *SD*=0.59) and if the tutor’s feedback was helpful (*M*=4.44; *SD*=1.09). Furthermore, N=12 students (75%) in the present study stated that they felt better prepared for such discussions and N=13 students (81.25%) stated that the skills lab was an opportunity to apply existing knowledge in practice. It was also shown that N=14 students (87.5%) rated the feedback from the SP as helpful. Moreover, N=15 students (93.75%) stated that they would like to complete another communication training in the skills lab. The assessments at the individual item level are shown in table 2 (Tab. 2).

In addition to the previous assessments, an open response format was used to record which aspects of the communication training the students particularly liked and which comments and suggestions for improvement could be used to further develop the training. A summary of the feedback can be found in table 3 (Tab. 3) and table 4 (Tab. 4). 

## 4. Discussion

The aim of the SRH University of Applied Sciences Campus Gera was to develop, test and evaluate communication training in a skills lab for the bachelor's degree programme in psychology. 

The psychology students rated their communicative self-efficacy higher after completing the communication training than before completing it. The results of the present study are thus in line with previous studies describing the positive influence of communication training on communicative self-efficacy in the fields of medicine and psychology [20], [26], [33], [34]. At the individual item level, a statistically significant improvement with medium to high effect sizes was recorded for eight of the 12 items on communicative self-efficacy. The students achieved the greatest improvement in their self-assessment for the items “successfully structuring the conversation” and “successfully identifying topics of conversation”. There was no statistically relevant improvement for the following items: “appropriate non-verbal behaviour”, “listen attentively without interrupting”, “encourage people to describe problems or worries” and “show empathy”. Encouraging the description of worries and problems probably requires several rounds to improve communicative self-efficacy. This is also recommended by Hartung [3], who argues that a one-time training session is not enough. For the other three aspects, it is noticeable that the pre-values are already in the high range, so that improvement may be more difficult due to a ceiling effect. Empathy, in particular, is already rated very highly by the psychology students, which is similar to the findings of Schochow et al. [35] for medical education students.

The communication training was evaluated positively by the students. All items received a positive rating from the students. Hence, the results support the assumption that skills labs enable early practical exposure [36] and that students benefit from the practice-based teaching in the skills lab [37]. The results also suggest that psychology students evaluate the communication training as a successful supplementary session, as almost all of them would like to have another communication training in the skills lab and desire more communication training.

The analysis of the constructive comments and suggestions for improvement indicates that all of the expectations were met for more than half of the respondents. Around a third of the participants stated that they would have liked to have been better prepared for the assignment. This could indicate a possible lack of confidence on the part of the students in such interview situations, as only one module lecture had taken place at the time of the communication training and the students had not yet completed a practical semester. An evaluation round was held with all of the participants (SPs, skills lab expert, tutor, subject coordinator) to expand on the suggestions for improvement regarding subsequent runs. The methodological weaknesses of the study were also highlighted and are listed below.

### 4.1. Limitations

The first methodological limitation of this pilot study is the timing of the survey. The study data was collected one to five weeks after the students had completed the communication training. This meant that there was a large time gap for the students between the communication training itself and participation in the survey, which may have distorted the answers due to possible memory effects. However, as it was on average only 7.9 days afterwards, the distortion can be estimated as low to medium (50% responded in the first three days). The retrospective assessment of the pre-values should also be criticised because it is assumed that there must have been an improvement due to the completion of training. This phenomenon is also referred to as a look-back error [38]. Furthermore, the subjectivity of self-assessments should be supplemented and compared with objective measures such as external assessment, checklists or video feedback [39]. It should also be noted that the communication training in the skills lab was only offered once. It can already be considered a success that medium to large effects were achieved by completing the training once. However, a sustained improvement in communication skills and the conviction that these skills can be successfully applied can only be guaranteed through cyclical training [3]. Future studies should therefore be conducted in a longitudinal design with at least three measurement points (pre-, post-, follow-up), as is done in the study by Nørgaard et al. [26], and include refresher options. In addition, a control group should be added and the sample size increased. 

## 5. Conclusions

This study shows by way of example that simulation-based training in a skills lab with trained SP is a suitable, positively evaluated method for increasing the communicative self-efficacy of undergraduate psychology students. This confirms that students benefit from practice-based teaching in the context of communication training in the skills lab. Communication training should therefore already be included in the bachelor's programme so that it can contribute to the mandated practice- and skills-based university teaching, which can be built upon further in the master's programme in clinical psychology and psychotherapy.

## Authors’ ORCIDs

- Sabine Rehmer: [0009-0003-8957-6638]
- Susanne Hardecker: [0000-0002-0419-9091]
- Dorothea Portius: [0000-0003-2608-1091]
- Sara Ramminger: [0000-0002-7295-1134]
- Maximilian Schochow: [0000-0001-7901-2335]
- Hiltraut Paridon: [0000-0002-8652-7350]
- Alexander Ernst: [0009-0009-8295-3887]

## Competing interests

The authors declare that they have no competing interests. 

## Supplementary Material

Task description; checklist for tutor; feedback list for SP; model of depression; role instructions for SP; role mask, only in German

## Figures and Tables

**Table 1:**
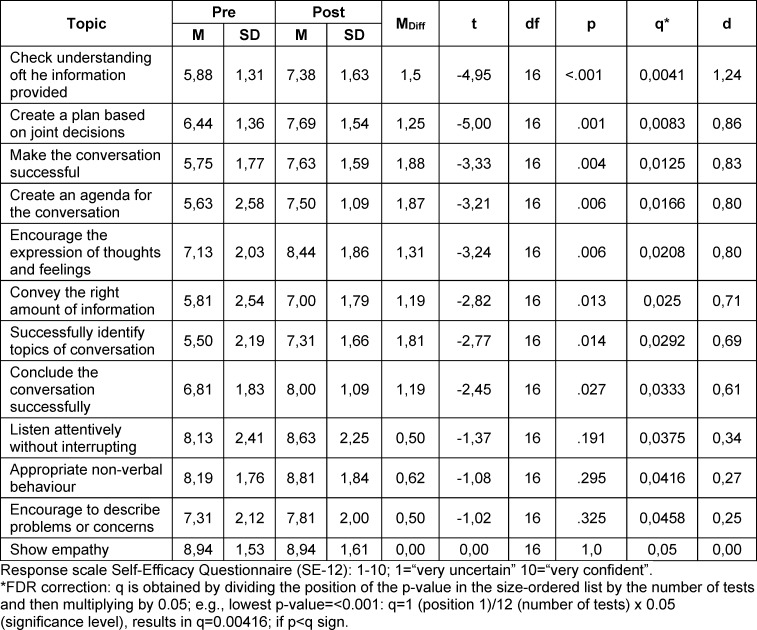
SE-12: Results of the t-test for dependent samples (by p-values in ascending order)

**Table 2:**
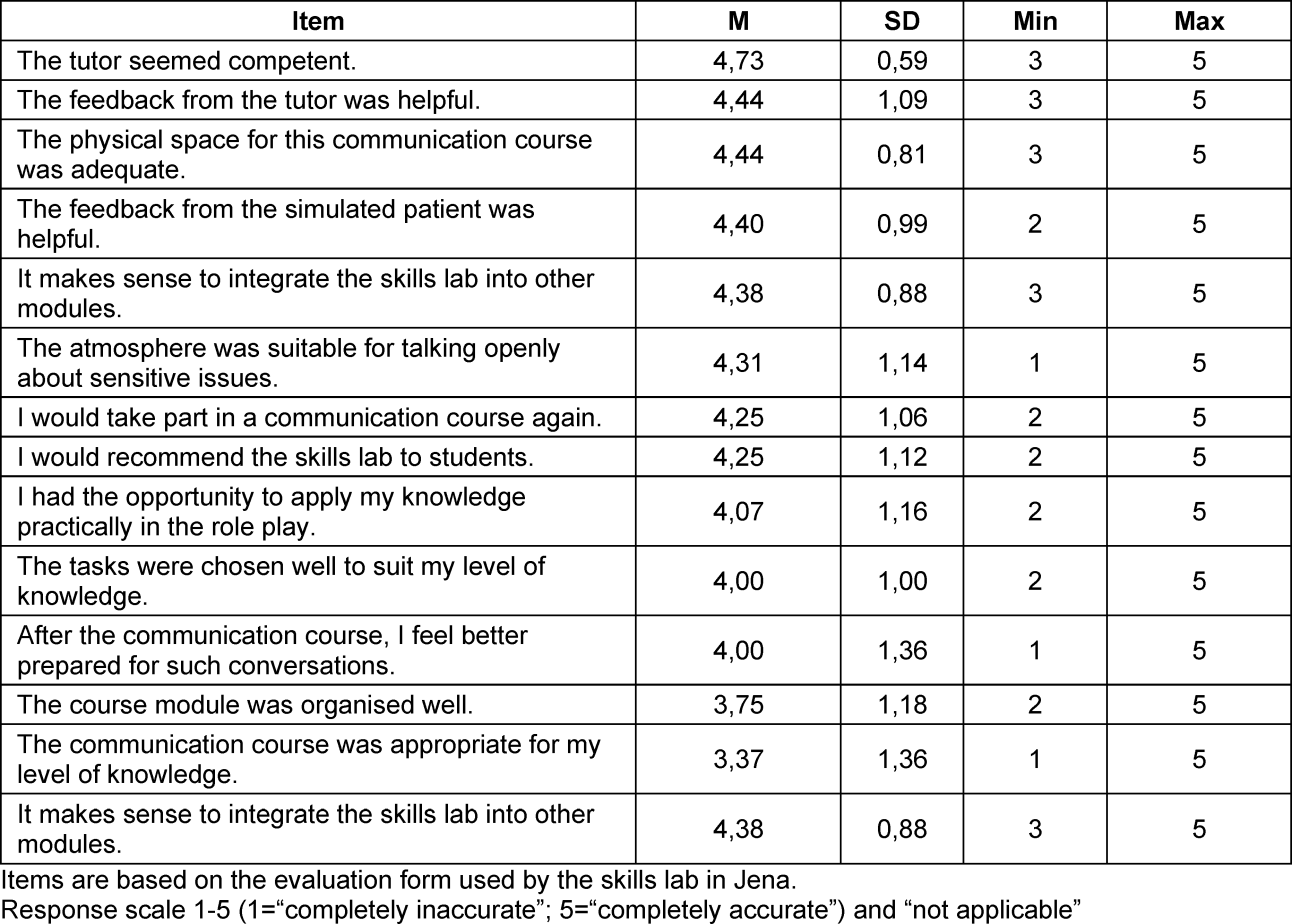
Results of the average calculation for the skills lab communication training assessment (in descending order)

**Table 3:**
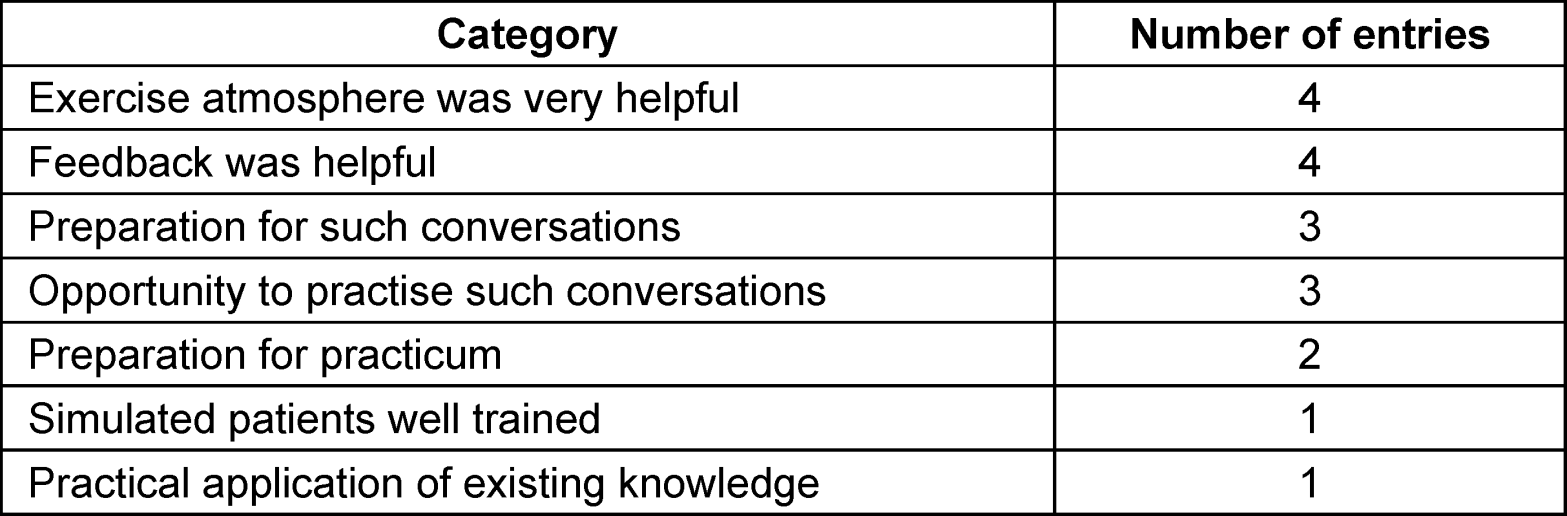
Descriptive evaluation of the item: “What did you particularly like about the communication training?” (multiple answers possible)

**Table 4:**
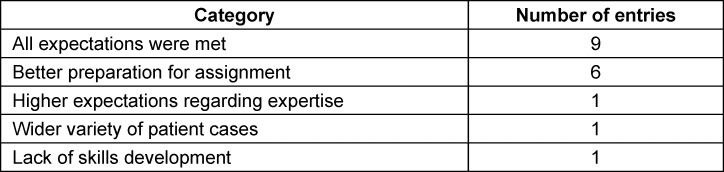
Descriptive evaluation of the item: “Which expectations regarding the communication training were not met?” (multiple answers possible)
