# Supplementary material for: Evaluation of skills lab communication training in a bachelor’s programme in psychology based on communicative self-efficacy
Source: GMS J Med Educ. 2025 Apr 15;42(2):Doc22. doi: 10.3205/zma001746 (PMC12131499; doi:10.3205/zma001746)
Supplement: Task description; checklist for tutor; feedback list for SP; model of depression; role instructions for SP; role mask, only in German [file JME-42-22-s-001.pdf]

Attachment 1: Task description; checklist for tutor; feedback list for SP; model of depression; role instructions for SP; role mask, only in German

Attachment 1 to Menzel M, Philipp S, Schulz K, Fankhänel T, Rehmer S, Haredecker S, Portius D, Ramminger S, Zergiebel U, SChochow M, Paridon H, Sänger S, Trummer A, Ernst A, Meusel S, Wick K. *An evaluation of skills lab communication training in a bachelor's programme in psychology based on communicative self-efficacy*. GMS J Med Educ. 2024;42(2):Doc22. DOI: 10.3205/zma001746

Informationen zum Aufklärungsgespräch
„Frau Müller ist antriebslos“

Situation:

Das Gespräch findet in einer psychosomatischen Rehaklinik statt. Hier wird Patient*innen geholfen zu einem strukturierten Tagesablauf zurückzukehren; es werden Stressbewältigungsstrategien sowie aktivierende Verfahren vermittelt. Neben dem Beziehungsaufbau findet im ersten Schritt der Verhaltensaktivierung die Psychoedukation statt.
Aus der Dokumentation des ärztlichen Aufnahmegesprächs entnehmen Sie die Informationen, dass Frau Carolin Müller unter depressiver Stimmung und Antriebslosigkeit leidet, sodass sie alltägliche Aufgaben nicht mehr bewältigen kann. Zudem ist eine Grübelneigung bekannt. Die von Carolin Müller beschriebene Symptomatik weist auf das Störungsbild einer Major Depression hin. Die Patientin ist motiviert und krankheitseinsichtig.

Ihre Aufgabe besteht in einer ausführlichen Aufklärung der Patientin über das Modell der Depression mit Fokus auf Verhaltensaktivierung.

Ihre Aufgabe:

Führen Sie ein Gespräch zur Aufklärung des Störungsbildes durch. Gehen Sie dabei auf die Entstehung und Aufrechterhaltung von Depressionen ein. Erarbeiten Sie gemeinsam mit Frau Müller das Teufelskreismodell.

Es geht in diesem Gespräch in erster Linie darum, das Modell ausführlich zu erklären, die Symptome der Patientin einzubinden und zu verdeutlichen, dass ein Verstärkerverlust vorliegt.
Gestalten Sie das Gespräch von der Begrüßung bis zur Verabschiedung.

Zeitrahmen: 20 Minuten (Zwei Minuten vor Ablauf der Zeit, erhalten Sie einen Hinweis durch den Tutor.)

Checkliste

	**Zu überprüfen:**	**Überprüft**
**1**	Tisch ist korrekt angeordnet	
**2**	Drei Stühle stehen im Raum	
**3**	Kameratechnik überprüft	
**4**	Kameratechnik positioniert	
**5**	Kopien zum Modell der Depression	
**6**	Kopien Aufgabenstellung	
**7**	Klemmbrett für Tutor zum Notizen machen während Gespräch	
**8**	Zeitkarte 2min (laminiert)	
**9**	Stoppuhr	
**10**	Stifte und Papier	
**11**	Raum wurde gelüftet	

Feedbackbogen SP

Datum:

	Positives	Verbesserungsvorschläge
**Begrüßung:** beide Namen, Handschlag, Platz etc.		
**Anlass** der Konsultation / Beschwerdevortrag		
**Metakommunikation**		
**Fragetechnik:** offene, geschlossene, Katalog, Suggestiv		
**Nonverbales:** Blick, Haltung, Stimme, Mimik, Gestik		
**Verbales:** Verstehen und Verständlichkeit, Fachsprache		
Ist der Umfang der erfragten **Informationen angemessen?**		
**Gesprächsstrukturierung**/-steuerung: bestimmt Themen: Unterbrechungen, Themenwechsel begründet, Pausen, Schweigen		
**Beziehung / Compliance**: Vertrauen, autoritär, partnerschaftlich, distanziert, symmetrisch, Übertragungsphänomene, Empathie, macht indirekte Vorwürfe, Schuld, Sympathie, erkennt Bedürfnisse des Patienten, Zusicherung von Unterstützung etc.		
**Gesprächsverhalten:** ermutigend, geduldig, unterstützend, Sicherheit ausstrahlend, nimmt mich ernst, beruhigend, engagiert, signalisiert Aufmerksamkeit, interessiert etc.		
**Umgang mit Zeit**		
**Schreiben:** erklärt warum		
**Informationsvermittlung:** verständlich, angemessen, widersprüchlich, vermeidet bestimmte Themen, Verstehensvergewisserung		
**Zusammenfassung** / **Therapievorschlag:** abgestimmt, einverstanden?		
**Anschlussfähigkeit:** Würde ich zu diesem Arzt / dieser Ärztin wiederkommen? Habe ich meine Ziele im Gespräch erreicht?		
**Struktur :**<br>**Beginn:** Begrüßung, Vorstellung, Klärung des Gesprächsthemas;<br>**Übergänge:** Metakommunikation (Kommunikation über die Kommunikation), Zusammenfassung des Gesagten;<br>**Ende:** Zusammenfassung des Gespräches, Verabschiedung		

Modell der Depression mit Fokus Verhaltensaktivierung (nach Martell et al., 2010)

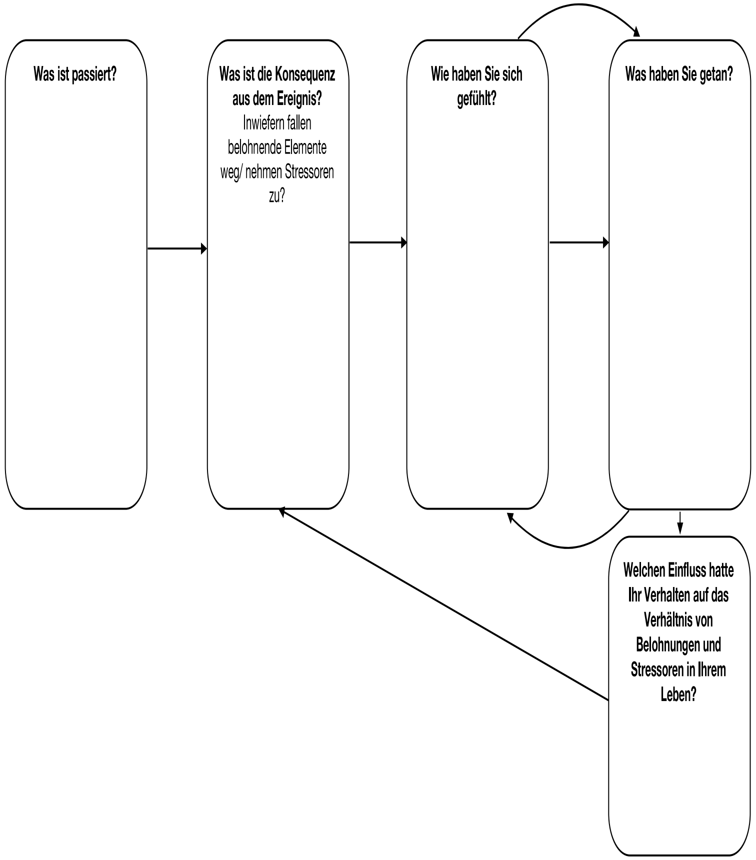

Rollenanleitung für Simulationspatient*innen

SkillsLab

Falltitel: „Frau Müller ist antriebslos“

Gesprächsart: Aufklärungsgespräch, Verhaltensaktivierung
Einbezug von Patienten in Behandlungsentscheidungen (Gemeinsame Entscheidungsfindung)

Allgemeine Charakteristika

Patientenname: Carolin Müller

Alter: 50 +-5

Geschlecht: weiblich

Muttersprache: deutsch

Aktuelle Rahmenbedingungen:
- Aktuelle Behandlung in psychosomatischer Rehaklinik
- Therapie auf Empfehlung der behandelnden Ärztin/ Arzt
- Erstgespräch

Symptomatik:
- in letzter Zeit fühlen Sie sich stark antriebslos
- Sie können kaum konstruktive Gedanken fassen
- grübeln viel
- Gedächtnisprobleme, Konzentrationsschwierigkeiten
- können sich an nichts mehr erfreuen
- Schlafen schlecht und sind tagsüber dann sehr erschöpft - dies macht sich im Alltag bemerkbar, denn Sie können Aufgaben nicht mehr effizient erledigen, ziehen sich vermehrt zurück
- Selbstvertrauen und Belastbarkeit sind stark reduziert
- Gespräche ebenfalls sehr anstrengend
- Passive Suizidgedanken
- Appetitverlust, bereits 4kg abgenommen

Biografie:
- kinderlos
- Sie hatten einen Lebenspartner für 4 Jahre - Trennung vor 6 Monaten
- Sie waren eigentlich immer sehr aktiv und sportlich - finden jedoch keine Kraft mehr dafür
- Abi auf zweitem Bildungsweg erkämpft - immer willensstark gewesen, viel Kraft geopfert für Job
- Job war sehr wichtiger Teil in Ihrem Leben (angesehene leitende Funktion in großer Firma), perfektionistisch, leistungs- und arbeitsorientiert
- Privatleben oft vernachlässig

- Arbeit verloren (vor 5 Monaten) - Arbeitslosigkeit, Geldverlust
- Fusion auf Arbeit aufgrund von Sparmaßnahmen, viele Jobs gekündigt - auch der eigene
- Sie sehen sich als Mobbing Opfer, sind verärgert über plötzliche Kündigung
- mit Kündigung nie gerechnet, da Sie immer sehr fleißig waren und gute Leistungen gezeigt haben
- fühlen sich ausrangiert und wertlos, nicht verstanden

Auslösende Situation:
- Verlust des Arbeitsplatzes
- Trennung von langjährigem Lebenspartner
- Summierung der Ereignisse
- Leistungsausgerichtetes Selbstkonzept, perfektionistische Ansprüche
- reagieren mit sozialem Rückzug, aktiviert depressives Schemata und dysfunktionales Gedankenkreisen um den Verlust des Arbeitsplatzes
- Kurzfristig Entlastung durch Rückzug
- Langfristig bleibt Depressivität bestehen
- Wenig Stressresistent

Aufgabe für den Schauspielpatienten:
Äußern Sie Ihre aktuellen Beschwerden und geben weitere Informationen auf Nachfrage. Fakten, die nicht erwähnt werden, gilt es zu improvisieren.
Lassen Sie sich das Modell der Verhaltensaktivierung erklären. Das aktuelle Auftreten ist freudlos jedoch kooperativ. Sie antworten teilweise zögernd und achten auf Ihre Körperhaltung.

Was der Studierende Sie fragen könnte	Wie Sie als Schauspielpatient*in antworten können
Wie geht es Ihnen heute? (zur Begrüßung, Einstieg)	Es geht so. Ich kämpfe mich durch den Tag und denke über so viele Dinge nach. Ich hatte auch schon wieder so starke Kopfschmerzen.<br>(Gerne Improvisieren)
Haben Sie Fragen zu dem Modell, was ich Ihnen gerade erklärt habe?	Nein, ich habe es ganz gut verstanden, weiß aber noch nicht so genau, was das mit mir zu tun haben soll.<br>Ja, was genau meinen Sie mit „Stressor“?<br>(Improvisieren: Je nachdem wie Sie das Gefühl haben, dass Erklärung für Sie nicht ersichtlich)
Was würden Sie an die Stelle eintragen *„Was ist passiert*?“ Was denken Sie, was der Auslöser ist?	Ich habe meine Arbeit verloren. Und davor habe ich mich von meinem Freund getrennt, aber mir ging es erst so schlecht nach der Kündigung.
Was resultiert aus diesem Ereignis? Welche belohnenden Elemente sind weggefallen?	Darüber habe ich noch nie nachgedacht. Ich habe kein Einkommen mehr und sehe meine Kolleg*innen nicht mehr.
Sind Stressoren hinzugekommen?	Geldprobleme, nichts zu tun, unterfordert, viele Bewerbungen.
Wie haben Sie sich dann gefühlt?	Sehr schlecht, ich konnte irgendwie nicht mehr lachen und ich war immer so müde und habe nichts mehr geschafft. Ich habe mich so wertlos gefühlt und enttäuscht.
Was resultierte aus diesen Gefühlen? Wie haben Sie sich verhalten?	Da muss ich kurz nachdenken. Ich glaube ich brauche einen kleinen Moment. Ich bin nicht mehr zum Sport gegangen und habe mich nicht mehr mit Freunden getroffen, dazu hatte ich einfach keine Kraft mehr, deshalb habe ich es einfach gelassen. Den Haushalt schaffe ich auch nicht mehr, früher hat das immer mein Freund gemacht.<br>Ich hatte auch ab und zu Suizidgedanken.
Was könnte Ihr Verhalten für einen Einfluss gehabt haben auf das Verhältnis zwischen Belohnungen und Stressoren?	Zu Hause geht eigentlich alles den Bach unter.
Sollte noch etwas ergänzt werden? Fällt Ihnen noch etwas ein?	Nein, mir fällt nichts ein. Ich bin mir unsicher.<br>Ich bekomme auch gerade schon wieder Kopfschmerzen.

Thema Verhaltensaktivierung:

- wenig komplex, schnell zu etablieren
- direkt an belastend empfundenen Symptomen ansetzen (bei Depression: sozialer Rückzug, Interessenverlust, Inaktivität)
- Stärkt Eigenverantwortlichkeit des Patienten
- Welche Verhaltensmuster gehen schlechter Stimmung voraus?
    - Kleine Verhaltensänderungen vereinbaren
    - Vermeidungsverhalten und wichtige Lebensziele identifizieren
    - Aktive Einbindung der Werte und Ziele der Person

Arbeitsblatt: Modell der Depression- Teufelskreismodell

Kurze Erklärung:

1. Was ist passiert? Auslöser identifizieren; Ziel ist es zu erkennen, dass Auslöser vielfältig sein können
2. Was sind die Konsequenzen? (Bsp.: mehr Stress, Schlafprobleme, weniger Freizeit, keine Routine) - hier entsteht Depression: Veränderter Zugang zu positiven Erlebnissen, hier wird sichtbar was sich eigentlich verändert hat.
3. Wie haben Sie sich gefühlt? (Ab hier Abwärtsspirale) Bsp.: Traurig, hoffnungslos, wertlos, verzweifelt.
4. Was haben Sie getan? Bsp.: Sozialer Rückzug, Schuldige suchen, mehr schlafen, Grübeln.
5. Auswirkungen: ich fühle mich nicht besser, sondern noch schlechter.

Ziel: Verdeutlichen, dass sich so die depressive Symptomatik verstärkt.

Rollenmaske

Geschrieben von: Maren Schneider / Dr. Swetlana Philipp
Lehrveranstaltung: Klinische Interventionen; VL 1: Verhaltensaktivierung

SkillsLab

Falltitel / Diagnose: Frau Müller ist antriebslos / Depression

Allgemeine Charakteristika:

1.	Fiktiver Patientenname:	Carolin Müller
2.	Alter:	50 +-5
3.	Geschlecht:	weiblich
4.	Sozialer Status / Ausbildung:<br>(z.B. leitender Angestellter, Schulabschluss, Weiterbildung, Studierende)	leitende Angestellte
5.	Muttersprache:<br>(z.B. deutsch, andere)	deutsch
6.	Persönlichkeitsmerkmale, Emotionen, Motivation und Einstellungen<br>(z.B. aufgeschlossen, zurückhaltend, aufdringlich, umgänglich, ängstlich, zwanghaft, höflich, schüchtern, unterwürfig, redselig, entscheidungsunfähig, überfordert)	gepflegte Erscheinung, wirkt still, introvertiert, freudlos (nur „gezwungenes" Lächeln), antriebslos.<br>Traurig, verzweifelt, … Sie neigen dazu, alles perfekt machen zu wollen und alles können zu müssen. Da Sie schlecht schlafen sind Sie sehr erschöpft und können alltägliche Aufgaben nicht mehr effizient erledigen.
7.	Interview-Typ<br>(z.B. Notfall, neuer Patient Allgemeinarztpraxis)	Erstgespräch in psychosomatischer Rehaklinik
8.	Ort und Zeit der Konsultation<br>(z.B. Praxis, Patientenzimmer)	Therapieraum
9.	Vorabinformationen über die Situation für „den Psychologen/die Psychologin":<br>(was die Studierenden über die Situation und den Patienten wissen)	Das Gespräch findet in einer psychosomatischen Rehaklinik statt, wo Patient*innen geholfen wird, zu einem strukturierten Tagesablauf zurückzukehren; es werden Stressbewältigungsstrategien sowie aktivierende Verfahren vermittelt. Neben dem Beziehungsaufbau findet im ersten Schritt der Verhaltensaktivierung die Psychoedukation statt.<br>Aus der Dokumentation des ärztlichen Aufnahmegesprächs entnehmen Sie die Informationen, dass Frau Müller unter unverhältnismäßig depressiver Stimmung und Antriebslosigkeit leidet, sodass sie alltägliche Aufgaben nicht mehr bewältigen kann. Zudem ist eine Grübelneigung bekannt. Die von Frau Müller beschriebene Symptomatik weist auf das Störungsbild einer Major Depression hin. Die Patientin ist motiviert und krankheitseinsichtig.

10.	Lernziel / Prüfungsziel:	Die Psycholog*in sollte:<br>- über das Vorgehen der Behandlung aufklären<br>- Aufklärung über Störungsbild Major Depression<br>- das Modell der Depression im Fokus auf Verhaltensaktivierung erläutern<br>- gemeinsam mit Patient*in dessen Symptome auf das Modell anpassen<br>- an individuelle Symptomatik anknüpfen können<br>- verdeutlichen, dass ein Verstärkerverlust vorliegt<br>- auf Rückfragen reagieren können
**Krankengeschichte**		
11.	Hauptbeschwerde, kurz und in den Worten des Patienten:<br>(Das können die Sätze sein, die der Patient im Wartezimmer sitzend vorbereitet hat, was er der Ärztin sagen möchte und/oder sein tatsächliches Problem)	*„Ich schaffe es nicht, morgens aufzustehen und den Haushalt zu bewältigen“*<br>- Sie leiden unter Appetitlosigkeit und können sich beim Einkaufen nie entscheiden. In den letzten Wochen haben Sie auch 4kg abgenommen.<br>- tagsüber sind Sie häufig sehr erschöpft und gehen früh ins Bett. Meistens können Sie trotzdem nicht gleich einschlafen und machen sich Vorwürfe, weil gerade überhaupt nichts klappt. Morgens würden Sie am liebsten im Bett liegen bleiben. Es fehlt Ihnen jeglicher Antrieb und Sie müssen Ihrem Körper „befehlen aufzustehen“.
12.	Leitsymptom und Begleitsymptom (bitte machen Sie zu allen Dimensionen möglichst genaue Angaben, ggf. zu jedem Symptom alle 7 Fragen erneut beantworten):	- HS: gedrückte Stimmung (sich traurig, niedergeschlagen, deprimiert fühlen - v.a. morgens)<br>- HS: Antriebslosigkeit<br>- ZS: Aufmerksamkeits- und Konzentrationsstörungen<br>- ZS: Schlafstörungen (Ein- und Durchschlafstörungen)<br>- ZS: Appetitmangel
	Zeitlicher Ablauf<br>(Beginn, Verlauf, Dauer, frühere Episoden)	- seit einigen Wochen<br>- die meiste Zeit des Tages, fast jeden Tag<br>- ist das erste Mal/die erste Episode
	Qualität<br>(Merkmale des Schmerzes)	- besonders schlimm am Morgen (Antriebslosigkeit)
	Begleitsymptome<br>(Was noch?)	- Kopfschmerzen
	Funktionsstörung<br>(Beeinträchtigung im Alltag)	- ich komme einfach nicht in Gang<br>- ich kann mich überhaupt nicht konzentrieren, da ich so viel grüble
	Kondition<br>(was hilft/verschlimmert; auch was bisher unternommen?)	- ich gehe weniger raus und nicht mehr zum Sport, da ich keine Kraft dafür habe
	Subjektive Vorstellung	- ich bin andauernd erschöpft, weiß aber nicht wovon<br>- ich weiß nicht, wie ich so krank werden konnte, kann es mir nicht erklären<br>- kein Konkreter Krankheitsauslöser bekannt

13.	Vegetative Anamnese<br>(vor allem Veränderungen interessant: Appetit/Ernährung, Trinkverhalten, Übelkeit, Erbrechen, Zittern, Gewicht, Miktion, Stuhlgang, Nykturie, Schlaf (Ein- und Durchschlafstörung?), Schwitzen/Nachtschweiß, Kopfschmerzen, Schwindel, Fieber, Alkohol, Nikotin, Drogen)	- Essen und Trinken wird vernachlässigt, Appetitmangel, Gewichtsverlust 3-4 Kg in den letzten 3-4 Monaten<br>- Schlaf seit einigen Wochen schlecht (kann schlecht einschlafen, weil ihr so viel durch den Kopf geht: *„ Ich muss den Haushalt schaffen“* / *„ Ich brauche einen Job“* / *„ Was, wenn ich mich weiter so schlecht konzentrieren kann, dann werde ich eh wieder gekündigt“*; wacht nachts häufig auf und kommt dann schlecht in den Schlaf; fühlt sich am nächsten Morgen nicht erholt und ist den ganzen Tag über müde und fühlt sich erschöpft)<br>- vermindertes Denk- oder Konzentrationsvermögen
14.	Familienanamnese<br>(direkte Verwandte) (genetische Erkrankungen, Herz-Kreislauf-Erkrankungen, Hypertonie, Krebserkrankungen, Diabetes mellitus)	- ob es in der Familie schon mal Depression gab, weiß der Patient/die Patientin nicht
15.	Sozialanamnese<br>(soziales Umfeld, Wohnverhältnisse, Partnerschaft, Arbeit, Beruf, Religion, Ressourcen)	- alleinlebend<br>- wenig Freunde<br>- soziale Isolation<br>- individuelle Gestaltung
16.	Sexualanamnese<br>(funktionelle Beeinträchtigungen, regelmäßige Untersuchungen (Gyn./Uro.), Schwangerschaft, (Fehl-) Geburten, Verhütungsmittel, Periode)	- zur Zeit keinen Partner
17.	Biografische Anamnese<br>(allgemein: wichtige oder den Patienten prägende Ereignisse)	- Abi auf zweitem Bildungsweg erkämpft- immer willensstark gewesen, viel Kraft geopfert für Job<br>- Job war wichtiger Teil im Leben der Patientin, perfektionistisch, leistungsorientiert<br>- Privatleben oft vernachlässigt
18.	Emotionale Situation des Patienten (Ängste und Sorgen) sowie die Einstellung zur Krankheit<br>(dazu gehört die subjektive Krankheitstheorie: woher kommen die Beschwerden und was könnte helfen?)	Frage nach Suizid: zunächst erschrickt die Patientin über die Frage… spricht nach einer Pause bzw. einem guten „Gesprächsangebot“ dann darüber, dass sie sich manchmal fragt, wie es wäre, wenn sie nicht mehr auf der Welt wäre.<br>- Wichtig: Latente suizidale Gedanken, aber keine akuten Handlungsabsichten oder Pläne, protektiver Faktor: die Familie *(„ das würde ich meiner Familie nie antun können“)*
19.	Typische Äußerungen oder Fragen des Patienten, die sich wiederholen in Textbausteinen (z.B. Wann kann ich wieder arbeiten? Werde ich daran sterben?)	- *„ Ich fühle mich einfach so nutzlos“*<br>- *„ Ich schaffe nichts mehr“*<br>- *„ So kann man mich nicht gebrauchen“*<br>- *„ Ich finde sowieso keine Arbeit mehr“*
20.	Körperhaltung und Körpersprache<br>(z.B. Hände reiben, Nägel kauen)	- häufig gesenkter Blick<br>- leise und monotone Sprache<br>- Nonverbal, im Denken und Sprechen verlangsamt

21.	Schwierigkeiten im Gespräch (z.B. bei Patienten, die indiskrete Fragen stellen oder viel reden, bitte ein Motiv für dieses Verhalten angeben)	- Es ist gut möglich, dass die Studenten mit der Situation überfordert sind. Vielleicht können Sie gegen Ende andeuten, dass allein das Gespräch Ihnen schon sehr gut getan hat und Sie gern wiederkommen möchten.
22.	Diese Daten werden vom Patienten nur erwähnt, wenn er / sie explizit danach gefragt wird (z.B. Sorge um alkoholkranken Vater)	- Sorge um Eltern, die bisher finanziell durch Sie unterstützt wurden<br>- Eltern in betreutem Heim lebend, benötigen viel Unterstützung

23.	Lebenslinie:<br>(Geburtsjahr, Jahr der Einschulung, Schulabschluss, Berufsausbildung/Studium, Familiengründung, Renteneintritt, usw.)
24.	Soziales Netzwerk:<br>(Freunde, Familie, Bekannte, Nachbarn, Kollegen – Intensität der Beziehungen?)
